# Dolphin-Assisted Therapy: Claims versus Evidence

**DOI:** 10.1155/2012/839792

**Published:** 2012-07-24

**Authors:** Britta L. Fiksdal, Daniel Houlihan, Aaron C. Barnes

**Affiliations:** ^1^Psychology Department AH23, Minnesota State University, Mankato, MN 56001, USA; ^2^School of Education, University of Wisconsin Stout Menomonie, WI 54751, USA

## Abstract

The purpose of this paper is to review and critique studies that have been conducted on dolphin-assisted therapy for children with various disorders. Studies have been released claiming swimming with dolphins is therapeutic and beneficial for children with autism, attention deficit hyperactivity disorder, physical disabilities, and other psychological disorders. The majority of the studies conducted supporting the effectiveness of dolphin-assisted therapy have been found to have major methodological concerns making it impossible to draw valid conclusions. Readers will be informed of the history of, theory behind, and variations of dolphin-assisted therapy along with a review and critique of studies published which purportedly support its use.

## 1. Dolphin-Assisted Therapy: Claims versus Evidence

Along with other pervasive conditions, those identified as having autism spectrum disorders (ASD) have often been subject to questionable or controversial treatments [1]. As defined by Simpson, a controversial treatment is any method or strategy that has not been validated by scientific support. Such treatments may be of special concern when seemingly extraordinary results are guaranteed. Such concerns may be confounded when physicians fail to anticipate or understand the feelings of desperation that accompany some parents of children with ASD when they come into the office [2]. The combination of desperation and a lack of effective treatment options provided by the physician may lead parents to pursue treatments with little or no empirical support. The number of diagnosed cases of ASD has increased ten times in the past 20 years with the current rate of one in every 166 children born being diagnosed [3]. It was estimated that in 2005 the National Institutes of Health spent $99 million on autism research. This number makes a stark increase compared to the $22 million spent in 1997 [3]. Taken together, there exist many opportunities for the eager pursuit of a wide range of treatments. Among those sharing relatively heightened interest and relatively little empirical support are animal-based treatments.

As noted by Morrison [4], for more than 12,000 years, animals and humans have been in therapeutic relationships together. Dogs are generally the most common therapeutic animal, but the literature and historic record suggests that cats, guinea pigs, cockatoos, African grays, horses, chickens, pot-bellied pigs, llamas, goats, and donkeys have all been utilized with therapeutic aims [4]. The rate of animal-facilitated therapy has increased dramatically over the past few years; however, the number of empirical research studies showing the efficacy of this therapy has not. Betsy Johnson was among the first to discover using dolphins as a therapeutic agent with individuals suffering from neurological impairments [5]. The grace and beauty of dolphins along with their responsiveness to humans have led therapists and researchers to assess potential therapeutic benefits. This interest, however, has taken a reckless turn and has led to the evolution of a treatment known as dolphin-assisted therapy (DAT) [6]. 

Dolphin-assisted therapies are primarily advertised through popular media such as television, informational movies, and the internet [7]. According to Marino and Lilienfeld [8], the claims made supporting DAT by the facilities themselves have not been empirically supported, neither has there been an increase in peer-reviewed papers published on the topic from the 1970s to 2007. Researchers and practitioners of DAT as well as parents with children diagnosed with an ASD should be aware that support for this type of treatment has not been empirically validated. Studies that have been held up as supporting DAT have serious methodological flaws rendering their results weak and meaningless [7–9]. Recent voices in the mainstream media have called for an aid in finding and selecting effective treatments, with the hope of reducing the number of parents chasing down the latest “fashionable” trends in intervention, and instead to base their decisions on valid data [3]. Unfortunately, organizations that have in the past been trusted for guidance, have had trouble avoiding affiliations with special interests [10]. Alternative treatments have sprung up, many with associated risk, with no empirical support and little documentation. However, many of these unconventional methods have the backing of major organizations supporting families with special needs children. 

This paper is an effort to navigate the often-spurious claims in the literature and popular media, and to increase the likelihood that those seeking effective treatment will be successful. The purpose of this paper is to provide a detailed description of DAT, review the studies published supporting DAT, review the studies that have debunked this particular treatment, and summarize the overall lack of empirical support for its use.

## 2. Overview of DAT

Dolphin assisted therapy has been used with the aim of treating individuals identified as having mental and physical disabilities for over 25 years [11]. DAT is a type of animal-assisted therapy that claims to help those who are physically and mentally ill and disabled as well as adults and children with various psychopathologies. Therapy generally involves the patient swimming and playing with dolphins in captivity-over several sessions while working on tasks such as hand-eye coordination or various verbal response targets. It is a highly attractive form of therapy due to the dolphins being well-liked, exotic animals [7, 8]. According to Nathanson et al. [12], the primary purpose of his DAT program is to increase engagement and target behaviors based on the child's individualized program by using dolphins to compliment or assist other, more traditional, treatments. The program focuses on increasing the frequency of target behaviors by using basic-behavior modification principles in a relatively short-term intensive therapy [13].

There are many different variations of dolphin-assisted therapy ranging from the client simply looking at or taking care of a dolphin, touching the dolphin, to entering the water and swimming with the dolphin. Different therapists have different theories on how humans and dolphins interact as well as the particular kind of therapy that should be employed for a specific patient [14]. The length and frequency of sessions vary depending on the program. Some therapists run sessions for a week, two weeks, or a month. Some programs have even tried single sessions that last a couple of hours instead of the typical 10–30 minutes [12]. Humphries [15] found that, in five of the six studies she evaluated, sessions lasted on average 30 minutes each and each study consisted of approximately 16 sessions total. 

In one example of a treatment plan, children first go through an on-dock orientation that consists of the therapist and child sitting on the edge of a 2-3-inch raised dock while the trainer is in the water manipulating the dolphin's movement. Children are typically able to touch, play, or give simple hand commands to the dolphins during this orientation to get them acquainted and comfortable with the dolphin. Once the child has completed the orientation stage, they start a series of therapeutic sessions. During these therapeutic sessions, children are allowed to play with the dolphins for a short time either from the dock or by going in the water with them after emitting a correct motor, language, or cognitive response. During the “play” time the children can touch or kiss the dolphin, dance in a circle with the dolphin, or ride on the dolphin by holding onto the dorsal fin [15]. 

Humphries [15] found the cost of DAT varies depending on the length and location of therapy as well as the therapy package chosen. There are currently DAT programs all over the world including Europe, the Middle East, Asia, USA, the Caribbean, Mexico, Israel, Russia, Japan, China, Bahamas, and South America [7, 14]. The typical price for five 40-minute sessions is about $2,600. Typical travel, food, and lodging costs can raise the price to $5,200 over two weeks. One notable example quoted in 2006, Nathanson's Dolphin Human Therapy, costs $7,800 for two weeks or $11,800 for three weeks not including travel, food, and lodging [15]. It is worth noting that these sums of money are being exchanged for activities that are often nearly indistinguishable from swim-with-dolphin programs typically frequented by tourists [7–9]. 

## 3. DAT Claims and Indicated Treatments

Nathanson [13] reported the two-week Dolphin Human Therapy program significantly increases language, speech, gross motor, and fine motor functioning among children with various disabilities when compared to the more conventional speech or physical therapy programs that last a minimum of six months. DAT has been targeted for children and adults of all ages, all genders, and all ethnicities [4]. Supporters and therapists of DAT claim it is effective in treating people with clinical disorders as well as conditions including autism, epilepsy, Angelman syndrome, Down syndrome, dyslexia, Rett syndrome, Tay-Sachs disease, Tourette syndrome, William syndrome, cancer, and AIDS [5, 7]. Other purported benefits of DAT include increased stimulation, better memory, increased motor skills, accelerated healing, and an increase in a person's well-being [5] as well as reduced stress, pain, and depression, increasing relaxation, enhancing the production of infection fighting t cells, endorphins, and hormones, and enhancing the recovery process [6]. In summary, Nathanson et al. [12] claim that DAT increases attention span, motivation, and language skills more rapidly and cost effectively than other more conventional therapies and the treatment effects are maintained over an extended period of time.

## 4. History of Dolphin-Assisted Therapy

What would come to be known as dolphin-assisted therapy dates back to the work of John Lilly in the 1950s [6]. His work was extended during the 1970s when dolphin researchers started studying interactions between dolphins and children with neurological impairments [15]. An educational anthropologist at Florida International University, Betsy Smith, is credited with conducting the first line of research in 1971 involving dolphins and children with neurological impairments [6, 7]. 

Since 1982, there have been only a small number of publications involving dolphin-assisted therapy [11]. Nathanson's first pilot studies on dolphin-assisted therapy took place at Ocean World in Ft. Lauderdale, FL, in 1978 and 1979. Based on the pilot studies, DAT was altered to consist of one session per day, two days per week with only one therapist for the program at Dolphin Research Center in Grassy Key, FL, from 1988–1994 [13]. According to Nathanson, his Dolphin Human Therapy program began providing full-time therapy across the years of 1995 and 1996. Sessions were offered five days a week and multiple therapists were employed at Dolphins Plus in Key Largo, FL. In 1997, a full-time, multiple therapist program opened in Miami, FL, at the Miami Seaquarium on Virginia Key. Between 1988–1997, children who entered dolphin-assisted therapy were diagnosed with over 40 different diagnoses and came from over 37 different states and 39 different countries. Many of the children were diagnosed with multiple disabilities. In 1997, Nathanson and colleagues started scheduling almost all of the children enrolled in Dolphin Human Therapy for at least two weeks of sessions instead of one [12, 13].

## 5. Theories behind Dolphin-Assisted Therapy

There have been numerous theories voiced regarding how dolphin-assisted therapy works. These theories are often presented to the public in verbose and vague language, using terms that sound technical but really have no significant meaning. When precise scientific terms are used, they are often used incorrectly or without proper context. Such obfuscating language is typically applied by pseudoscience practitioners with the purported aim of sounding more advanced, legitimate, and scientific [5]. Since the exact etiology of autism is unknown, advocates of DAT are able to create any explanation for the efficacy of the treatment. There are no limits or regulation on the number of erroneous claims that can be made [7]. The three most prominent theories for DAT are echolocation, dolphins being attracted to people with disabilities, and overall joy and relaxation. Other theories include simply being in the water and increasing attention in individuals with autism [6–9, 15].

Nathanson [13], Brensing et al. [11], McKinney et al. [6], and other proponents of DAT have claimed that ultrasound emitted by dolphins through echolocation clicks has a mechanical effect on human endocrine and neural systems. These effects enhance healing by changing the individual's body tissue and cell structure. This is one of the most popular theories behind DAT; however, the evidence backing these claims appears to be purely anecdotal [6]. Dolphins produce sounds, often described as clicks, and below the blowhole as part of a technique called echolocation dolphins can emit a rate of 300 clicks per second, using echolocation to navigate, find food, and communicate with other dolphins [6]. Simply put, a human interacting with dolphins differs from the current standards of medical practice for therapeutic ultrasound, which call for repeated application at a specific intensity and duration [11]. 

Other theorists propose that dolphins are sensitive to people with disabilities and that they seek to help them by paying extra attention to them through playful expressions of concern [6]. This has been termed “secret language” by some DAT therapists. In the 70s, Dr. Smith theorized that dolphins could communicate acoustically with body movements and are attended to the body movements of others. This appeared to be especially true in the case of children with autism. It seemed to DAT's supporters that the dolphins understood their thoughts and actions [6].

It has also been theorized that through DAT, human contact with dolphins produces intense emotions and feelings of reconnection and happiness which consequently increases the well being of the participant [5]. Dolphins have been reported to bring joy and happiness to people through their playful behavior and constant “smile.” The joy, novelty of the situation, and extra attention are likely components that enhance a person's quality of life thus increasing the motivation to learn [6]. It may be that dolphins become positive reinforcers for the patient emitting a specific behavior or achieving a therapy goal [7]. For some people, interacting with animals in general has a calm and stress-reducing effect. Brensing et al. [11] found that dolphins have a relaxing influence on people based on the analysis of EEG scans.

In addition to ultrasound-based theories, Nathanson based his dolphin-assisted therapy on the theory that, as a result of swimming with dolphins, children will increase their attention to stimuli in the environment [15]. Nathanson's attention deficit hypothesis implies that people with mental retardation and other disorders are unable to learn because of a deficit in physiological attention to the important details of the stimuli and not because they are unable to process information. This contributes the overarching theory that animals increase attention for individuals, therefore leading to improved cognitive processes such as enhanced learning, motor skills, language, and memory [13]. 

McKinney et al. [6] report that simply being in water has a relaxing therapeutic effect on people with various disabilities. Aquatic therapists claim that adding the calming effects of animals such as dolphins to being in water will enhance the therapeutic effects.

## 6. Research behind Dolphin-Assisted Therapy

There have been multiple claims made supporting dolphin-assisted therapy. Lukina [16] as well as Servais [17] claim that DAT improved language, cognitive processing, attention, behavior, motivation to learn, and even some medical conditions. Nathanson [13] and Nathanson et al. [12] claim that dolphin human therapy has successfully increased motivation, gross and fine motor skills, speech, language, and attention. They also claim that two weeks of therapy is just as good if not better than six months of other traditional treatments. Humphries [15] evaluated six studies supporting DAT [12, 13, 16–19] and found all of the studies were at risk for investigator bias, novelty of the therapy, and multiple treatment interference. 

Morrison [4] found that there were several methodological weaknesses consistently seen throughout DAT studies. Some of these weaknesses included lack of consistent randomization of participants, small sample size, absence of control or typical care group, lack of reliability and validity measurements, attrition rates, poor generalization, selection bias, and novelty effect. According to Marino and Lilienfeld [8], if these methodological weaknesses were accounted for, it would be shown that there is nothing unique or special about dolphins per se in DAT. 

Dolphin-assisted therapy receives the majority of its advertisement, praise, and positive attention from the media (e.g., news programs, promotional films). Some reports claim the success rates for physical and behavioral improvement through DAT is 90% [15]. Marino and Lilienfeld [7] found one website that claimed, “The field of medicine has shown extraordinary results of the therapy (DAT) and breakthroughs in outcomes in relation to conventional methods of treatments such as prescribed medication, human therapy, and others.” The popularity for DAT continues to be substantial, while the research base continues to be meager at best [8, 15]. 

In 2003, Dr. Betsy Smith, one of the first researchers to investigate and propose the possibility of DAT having therapeutic value, denounced its use. Describing it as an ineffective and exploitative practice, Dr. Smith voiced two main concerns: (1) monetary gain was more involved with DAT's practice than was empirical evidence supporting its use, and (2) it was undermined and detracted from valid therapy programs [7]. Purveyors of DAT programs can expect to gain a substantial amount of money from every family and client who seeks their help. The potentially hazardous impact of time and money spent for DAT is compounded when the same resources could be spent on empirically supported treatments (e.g., discrete-trial teaching). Currently, there are no studies that show DAT to be consistently effective [5]. 

Nathanson et al. [12] argued that compared to conventional long-term therapies, Dolphin Human Therapy, a form of DAT, achieved effective results more quickly and at lower cost. Nathanson and colleagues compared two weeks of DAT to six months of speech and physical therapy with individuals with multiple disabilities. Each participant received six months of conventional therapy right before DAT and had received 16 or 17 sessions throughout a two-week DAT program. They claimed the administration of DAT to children with severe disabilities significantly increased motivation, motor skills, attention, and language. Results showed that prior to DAT 0% of the children were able to make the independent target response and after DAT 57–71% were able to, therefore making an argument supporting DAT as an effective treatment for individuals with severe disabilities [12].

 In 1998, Nathanson conducted another study examining the long-term effects of DAT and found the increases in functioning were maintained or improved at the one-year followup. Nathanson sent out 137 questionnaires to assess the long-term effects of DAT, of which 71 were returned (52%). Following DAT, it was expected that children would return and continue with their conventional therapies such as occupational, speech, and physical therapies, parent follow ups, and special education services. According to the questionnaires that were returned, following DAT clients increased the amount of time they participated in and benefited from their conventional therapies more than 50%. This study also found that two weeks of therapy were significantly more effective than one week and that their model of DAT, Dolphin Human Therapy, showed beneficial long-term effects for approximately 95% of the children treated [13].

Marino and Lilienfeld [9] found multiple reasons why both studies, Nathanson et al. [12] and Nathanson [13], should be interpreted with caution. After assessing both articles, a minimum of 11 methodological weaknesses were found that undermined the scientific validity for both studies. The main weaknesses consisted of the potential for placebo effect, history effects, and regression to the mean all due to a lack of experimental control [7].

The study conducted by Nathanson and colleagues in 1997 appeared to have used a modification of a pre- postdesign. The “pre-test,” or selection criteria, was the fact that children were only able to participate contingent on their inability to respond on their own to a physical or verbal task. After two weeks of DAT, each child was assessed (the posttest) for their ability to respond on their own to the same task as before. Changes in response from pre- to posttest were attributed solely to DAT. Nathanson claimed to have used a single-subject design, however, he failed to report any individual subject's data. Instead, he compiled all the data from each subject into tables obscuring analysis at an individual level. Because of this aggregation of data, it is possible that some children did worse after therapy but the data was embedded in a group of children who did show improvements [9, 15].

The most important and detrimental flaws to the study conducted by Nathanson et al. in 1997 stem from a lack of experimental control. Lack of experimental control makes it impossible to attribute any changes to DAT alone. There was no control group for which to compare the treatment group, no dismantling strategy to expose subjects to the different treatment components in a systematic manner, and no counterbalancing between what they called the pre- and post- tests. Without a control group, it is impossible to rule out a placebo effect, regression to the mean, novelty effects, history, the effect water could have had on the children's performance, or other variables such as an increase in interpersonal attention or interpersonal contact. Nathanson et al. appear to have changed the way the dependent variable was assessed at various points in the study. This suggests that instrumentation might have been the cause of changes that appeared to occur. Many of the participants came from different states or even different countries. Nathanson and colleagues did not have a randomized control group to assess for variables associated with these children being in a different state and possibly even a different country. 

Another set of problems with the Nathanson et al. [12] study stems from how the children's responses were recorded and measured. Experimenter expectancy could have had an effect on the behavior recorded since the observers were aware of the outcome desired for the study. To add to the threat of experimenter expectancy, there was no operational criterion or definition differentiating between what was considered an independent response from one that was assisted or guided. Having a strong interrater reliability coefficient or a report on procedural integrity may have helped to minimize this methodological flaw; however, the authors never explained how they get their inter-rater reliability coefficient of 1.00 or what it represented. It could have been based on every trial from the entire study, it could have been a small sample of trials, or it could have only included trials in which there was perfect inter-rater reliability [9]. 

The follow-up study conducted by Nathanson in 1998 fell victim to many of the same methodological flaws as the 1997 study (i.e., history, placebo effects, instrumentation, lack of control group, and regression to the mean). Nathanson's follow-up data were based solely on a questionnaire filled out by the parents of the children who underwent DAT the year before [9]. Nathanson concluded that the children maintained their skills one year later, two weeks of DAT was better than one week, and there were no differences in long-term effects of DAT due to the participant's disorders(s) [13]. Nowhere in his paper did Nathanson attribute any of the changes to the months of conventional therapy each child had received in between the end of DAT and the follow-up questionnaire as well as in between the pre- and post- test measures [7–9].

Nathanson failed to control for demand characteristics, including the tendency for participants to respond in a way they feel is appropriate for what they think the researcher wants to hear. Not only did he fail to control for it, he made it worse by beginning each item of the questionnaire with a statement that attributes all success to dolphin-assisted therapy. This made the hypothesis of the researcher evident to each parent as they were filling out the questionnaire. The questions in the questionnaire only asked about the positive effects of DAT (mostly behaviors that were improved or maintained) and did not assess or ask about behaviors that might have gotten worse or regressed. Of the 137 questionnaires sent out, 71 were returned, and the study incorporated no pre- postmeasurement of parents' perceptions of behavior [9]. 

In another study, Lukina [16] assessed the effects of DAT on the psychoneurological functioning of children with various conditions compared to healthy children through the use of a single group pretest-posttest design. The participants included 30 children with infantile neurosis, 25 children with mental retardation and autism, 35 children with other unspecified diseases, and a comparison group of 57 children without major diagnoses. Each child interacted with the dolphins by swimming with them for 10–15 minutes for 5–10 sessions. The results indicated that the cardiac rhythms for each group increased after having swum with the dolphins. Lukina claimed that the results of the study supported the fact that the redistribution of “psychoemotional” dominants opens possibilities for psychotherapy and rehabilitation measures [16].

One major flaw in the Lukina's study was a lack of clear definition for “psychoemotional” dominants or how they are related to changes in cardiac rhythms. Furthermore, Lukina claimed that DAT reduced depression, night phobias, hysteria, and enuresis for the children in the “infantile neurosis” group, however, there was no data reported showing this was the case. Lukina also failed to mention the assessment instruments used to assess depression, night phobias, hysteria, and enuresis for these children. Psychotherapy was also a part of therapy, so attributing all the positive effects to DAT is impossible because the different treatment components were never assessed independently. Although there was a comparison group of children without major diagnoses, there was no control group of children who did not swim with dolphins. When you add to this the use of a single A-B comparison design lacking experimental rigor, there is clear reason to question the validity of the study [8, 15].

The study conducted by Servais [17] involved two experiments. The first experiment included two control groups (a classroom group and a computer group) and an experimental group (dolphin group). Each group consisted of three children with autism. Children from each group were taught the same cognitive task in their respective settings. The second experiment consisted of a dolphin group and a classroom group only. All groups in each experiment were given pre-tests first, followed by 10–15 “learning sessions” in which the cognitive tasks were taught in each of the groups, followed by each group being administered a post-test. Results showed the children working with the dolphins responded correctly more often compared to the children in the control groups. Outcome measures of social-emotional status revealed increases of kindness, attentiveness, initiating play, self-control, and eye contact were found with the children who participated in the DAT compared to the control groups. 

Servais [17] is also hindered by methodological and practical flaws that call validity into question. At times during the study, in order to increase exposure to the dolphins, the human subjects were provided with correct answers to the cognitive tasks [15]. According to Marino and Lilienfeld [8], Servais did not explicitly state whether the pre- and post-tests were the same within or across groups making it impossible to rule out instrumentation effects. The children in the first dolphin group improved and performed significantly better than those in the second dolphin group but no other differences were reported between groups in the first experiment. The children from the second dolphin group did not appear to have improved or performed significantly better than the control group from the second experiment. Other threats to the validity of the study include experimenter expectancy and demand characteristics because the author was the only person who coded the behavioral outcomes. It is unclear whether other components of therapy, (e.g., swimming outdoors, etc.) might have contributed to a child's improvement.

Likura et al. [19] looked at the effects of DAT on patients with atopic dermatitis (skin condition). There were two groups of patients, one group included swimming with dolphins in the seawater therapy, and the other group received only seawater therapy. For six days the patients swam with dolphins in seawater, which is typically painful for individuals suffering from atopic dermatitis. Dramatic skin changes have been reported after contact with seawater, however, patients typically complain of pain or stress making it difficult to stay in the water for prolonged periods. The purpose of the dolphins in this study was to minimize the stress and pain felt by the patients, thus distracting them and helping them to relax. Each DAT session lasted 90 minutes and each participant had two sessions per day for 6 days. Skin conditions for patients in both groups improved; however, the psychological well-being (level of pain and stress experienced) of the group that swam with dolphins was significantly better than the group without dolphins [8, 19].

 The main criticism of the study conducted by Likura and colleagues [19] is the lack of details given regarding what seawater therapy for patients with atopic dermatitis is. Along with a lack of details about the therapy, methodological components of the study were missing as well. It is impossible to attribute the positive effects on the stress and pain levels of the patients to swimming with dolphins when no information is given informing the reader what the non-DAT therapy entailed. The conclusions made by the authors are subjective and vague at best [8]. 

A study conducted by Antonioli and Reveley [18] assessed the effects of swimming with dolphins on the levels of depression and anxiety among individuals with mild to moderate depression. A control group consisting of individuals with mild to moderate depression was used to compare the effects of DAT on the experimental group. All subjects were required to quit taking any medications (antidepressants) or receiving psychotherapy four weeks before starting the study. A modified version of the Hamilton Rating Scale for Depression, the Beck Depression Inventory, and the Zung Self-Rating Anxiety Scale were used as behavioral and psychological measures during baseline and at the end of treatment. Results showed that individuals who were able to swim with the dolphins in the water reported significant improvement in the depression scores compared to the control group who swam in the water without dolphins.

According to Marino and Lilienfeld [8], the study conducted by Antonioli and Reveley [18] controlled for more extraneous variables when compared to any of the other studies conducted on DAT. Researchers randomly assigned individuals to control and experimental groups, they utilized pre- and post-tests with blind raters, and incorporated validated assessment instruments. Despite this, Marino and Lilienfeld found many limitations to the study. First, the participants were not blind to the condition making it impossible to rule out demand characteristics [7–9, 18]. Second, there was nothing done to control for possible placebo or novelty effects of interacting with an exotic animal. Third, because the study relied on self-report measures and the participants were not blind to the condition, informant bias is impossible to rule out [7–9]. Fourth, no follow-up study was conducted meaning that the differences between the control and experimental groups can only be explained by the different conditions at the time of the test [7–9, 18]. Fifth, nothing was done to control for “resentful demoralization” which refers to when a participant realizes they are receiving a less beneficial treatment and becomes resentful, thus potentially threatening construct validity [7–9]. According to Antonioli and Reveley they controlled for “resentful demoralization” by allowing the participants in the control group to swim with the dolphins after the final evaluation. However, since it occurred after the final evaluation there is no reason to believe “resentful demoralization” was not a threat [7–9]. 

## 7. Summary and Recommendations

In general, DAT is subject to criticisms regarding novelty due to the fact that dolphins are charismatic, exotic animals that most people will not regularly encounter in their daily lives. Future research should focus on reducing the novelty of dolphins by incorporating exposure to build familiarity prior to intervention or by using a comparison group that interacts with some other exotic, charismatic animal. Construct validity is consistently threatened when researchers fail to recognize that there are multiple components to a specific treatment. In the case of DAT, swimming in the water, being somewhere warmer, being in a different country or somewhere new, and sleeping and living in novel settings (e.g., hotel) are all potentially confounding variables that need to be controlled for in order to attribute changes solely to DAT. Construct confounding is generally controlled for by taking apart the treatment and testing each component separately through use of extended, multiple phase designs and control groups [8]. 

Despite these persistent threats to validity and the lack of empirical support for DAT, it is not surprising that many health professionals have continued to offer such treatment as an option. Likewise it is not surprising that those seeking treatment continue to heed the recommendations of both health professionals and the media to employ purveyors of DAT. McWilliam [20] found multiple reasons why people adopt unproven practices, including the following; many proven practices are more difficult to implement than unproven therapies, unproven practices sometimes reinforce the specialization of a professional, professionals tend to believe what other professionals tell them without investigating for themselves, people often tend to believe the results and research that support their established values and beliefs, many professionals do not have time to keep up reading the literature available on all relevant topics and research, and parents have ample motivation to serve as a source of hope and optimism in the face of the challenges their children are facing. Unproven therapies often claim to provide rapid results that are all encompassing (e.g., promising to return a child with autism back to their “normal” self) [1].

It is becoming more evident that reliance and unrestricted use of unproven therapies for children with autism are hindering the field of ASD treatment and research. A pattern of reliance on suspect therapies has led to unhealthy and unrealistic expectations for progress and improvement for children with ASD. Turk [21] calls for therapies to be held responsible for providing solid, empirical evidence for their use. DAT indicated for several conditions, including ASD, is an excellent example of the urgent need for clear, verifiable, and repeatable evidence within psychology supporting therapies. Specifications regarding cost effectiveness and efficacy of the treatment should be required before therapies are endorsed or supported. Overall, research studies need to be better designed and threats to validity must be addressed before we deem DAT as an effective intervention for any population [15].

## References

[1] Simpson RL (2005). Evidence-based practices and students with autism spectrum disorders. *Focus on Autism and Other Developmental Disabilities*.

[2] Harrington JW, Patrick PA, Edwards KS, Brand DA (2006). Parental beliefs about autism: implications for the treating physician. *Autism*.

[3] Kalb C (2005). When does autism start?. *Newsweek*.

[4] Morrison ML (2007). Health benefits of animal-assisted interventions. *Complementary Health Practice Review*.

[5] Marine Connection: Protecting Dolphins and Whales Worldwide. (n.d.) Truth about dolphin assisted therapy. http://www.marineconnection.org/campaigns/captivity_dat2006.html.

[6] McKinney A, Dustin D, Wolff R (2001). The promise of dolphin-assisted therapy. *Parks and Recreation*.

[7] Marino L, Lilienfeld SO (2007). Dolphin-assisted therapy for autism and other developmental disorders: a dangerous fad. *American Psychological Association*.

[8] Marino L, Lilienfeld SO (2007). Dolphin-assisted therapy: more flawed data and more flawed conclusions. *Anthrozoos*.

[9] Marino L, Lilienfeld SO (1998). Dophin-assisted therapy: flawed data, flawed conclusions. *Anthrozoos*.

[10] Tribune Watchdog Dubious Medicine NovAutism treatments: Risky alternative therapies have little basis in science. http://www.chicagotribune.com/health/chi-autism-treatments-nov22.0.1396079.story.

[11] Brensing K, Linke K, Todt D (2003). Can dolphins heal by ultrasound?. *Journal of Theoretical Biology*.

[12] Nathanson DE, de Castro D, Friend H, McMahon M (1997). Effectiveness of short-term dolphin-assisted therapy for children with severe disabilities. *Anthrozoos*.

[13] Nathanson DE (1998). Long-term effectiveness of dolphin-assisted therapy for children with severe disabilities. *Anthrozoos*.

[14] Dolphin Therapy(n.d.) http://www.researchautism.net/interventionitem.ikml?print&ra=64&infolevel=4.

[15] Humphries TL (2003). Effectiveness of dolphin-assisted therapy as a behavioral intervention for young children with disabilities. *Bridge*.

[16] Lukina LN (1999). The effect of dolphin-assisted therapy sessions on the functional status of children with psychoneurological disease symptoms. *Fiziologiia Cheloveka*.

[17] Servais V (1999). Some comments on context embodiment in zootherapy: the case of the Autidolfijn project. *Anthrozoos*.

[18] Antonioli C, Reveley MA (2005). Randomised controlled trial of animal facilitated therapy with dolphins in the treatment of depression. *British Medical Journal*.

[19] Iikura Y, Sakamoto Y, Imai T (2001). Dolphin-assisted seawater therapy for severe atopic dermatitis: an immunological and psychological study. *International Archives of Allergy and Immunology*.

[20] McWilliam RA (1999). Controversial practices: the need for a reacculturation of early intervention fields. *Topics in Early Childhood Special Education*.

[21] Turk J (2005). The developmental psychiatry manifesto. *Clinical Child Psychology and Psychiatry*.

